# Enhanced recovery after surgery (ERAS) relieves psychological stress in patients with osteoporotic vertebral compression fracture undergoing percutaneous kyphoplasty: an observational retrospective cohort study

**DOI:** 10.1186/s13018-023-03703-x

**Published:** 2023-03-20

**Authors:** Zhong-wei Ji, Chun-yang Fan, Zi-lin Yu, Xie-xing Wu, Hai-qing Mao

**Affiliations:** 1 Department of Orthopedic Surgery, The First Affiliated Hospital of Soochow University, Suzhou, 215006 Jiangsu China; 2 Department of Pain Management, Zhejiang Provincial People’s Hospital, People’s Hospital of Hangzhou Medical College, Hangzhou, Zhejiang China

**Keywords:** ERAS, PKP, OVCF, Depression, Anxiety

## Abstract

**Study design:**

This is an observational retrospective cohort study.

**Objective:**

The purpose of this study is to investigate the incidence rate of depression and anxiety and the changes in patients treated with percutaneous kyphoplasty (PKP) following ERAS protocol.

**Summary of background data:**

The incidence of depression and anxiety is not uncommon in patients with osteoporotic vertebral compression fracture (OVCF), which affects the prognosis of surgery. Enhanced recovery after surgery (ERAS) protocols can improve the perioperative stress response of patients.

**Materials and methods:**

Patients were treated conventionally in 2019 as the control group (CG) (n = 281), and patients were treated according to the ERAS protocol in 2020 as the intervention group (IG) (n = 251). All patients were evaluated for depression and anxiety using Patient Health Questionnaire-9 (PHQ-9) and Generalized Anxiety Disorder-7 at admission, postoperative 1 week, 1 month and 3, 6, 12 months.

**Results:**

The degree of depression statistically decreased in the IG at follow-up periods (*p* < 0.001), and the degree of anxiety statistically decreased at 1 week (*p* < 0.001), 1 month (*p* < 0.001), 3 months (*p* = 0.017). Patients in the IG could soothe depression and anxiety disorders faster than patients in the CG and maintain psychological stability at the follow-up periods. The percentage of moderate or above depression in the IG was statistically fewer than in the CG at follow-up periods (*p* < 0.01). The odds ratio (OR) was respectively 0.410, 0.357, 0.294, 0.333, 0.327 from 1 week to 12 months. While the percentage of patients with moderate or above anxiety significantly decreased in the IG at 1 week (*p* < 0.001), OR = 0.528, 1 month (*p* = 0.037), OR = 0.309 and 12 months (*p* = 0.040), OR = 0.554, no differences between 3 months (*p* = 0.187) and 6 months (*p* = 0.133).

**Conclusion:**

PKP following ERAS protocol to treat patients with OVCF had a better effect on relieving postoperative anxiety and depression than following conventional protocol.

## Introduction

Osteoporotic vertebral compression fracture (OVCF) is one of the most common and severe complications of osteoporosis, resulting in back pain, spinal deformity, decreased quality of life, and increased mortality [1]. Vertebral augmentation surgery, such as percutaneous kyphoplasty (PKP), has been proven to be an effective minimally invasive surgical treatment for OVCF [2]. Compared to conservative therapy, PKP allows patients to get out of bed earlier and avoid the problems of prolonged bed rest [3]. However, surgical complications cannot be ignored due to the perioperative stress response and the degeneration of the body functions of elderly patients.

In addition to physiological damage, psychological and social aspects are always affected in vertebral fracture patients, which appears to be associated significantly with depression and anxiety [4]. Clinical data indicated that morphometric vertebral fractures could affect the emotional pleasurability of patients and may exacerbate the disease even without clinically symptomatic vertebral fractures [5]. A review of studies reported poor mood and anxiety disorders are associated with an increased risk of osteoporosis [6]. Moreover, mental health can also be adversely impacted by fracture-related cardiovascular, pulmonary, and digestive complications and further affect patients] quality of life [7]. In terms of surgery, psychosocial factors have been the predictors of the efficacy, depression and anxiety could lead to various negative results [8–10]. Therefore, how to alleviate psychological problems seems to be an issue that cannot be ignored for surgical patients.

An evidence-based perioperative care protocol in surgery has been systematically applied called “enhanced recovery after surgery (ERAS)”, which aims to optimize health by reducing the surgical stress response through multidisciplinary collaboration, such as surgery, anesthesia, nursing, and nutrition [11]. Colorectal surgery, which put ERAS into practice earliest, offered a mass of literature supporting its benefits [12]. ERAS has been implemented in various surgical departments and effectively promoted patient recovery [13]. Psychological assessment and intervention, which seeks to reduce the anxiety and emotional burden of suffering from trauma and surgery, is an indispensable part of ERAS [14]. Depression and anxiety in spine surgery could lead to a more severe pain experience and exacerbated self-reported disability [15], which has been a great challenge we need to face and deserves more attention.

However, there is a lack of relevant studies on the psychological state of patients undergoing PKP. In this study, we investigated the incidence rate of depression and anxiety and the changes in patients treated with PKP following ERAS protocol.

## Methods

### Patient

The authors have established a registry of patients who have undergone PKP surgery. A convenience sample of patients was recruited from January to December in 2019 at the department of orthopedics, the first affiliated hospital of Soochow university as the control group (CG), while patients from January to December in 2020 as the intervention group (IG). The inclusion criteria were as follows: (1) older than 50 years and diagnosed osteoporosis by DXA; (2) a single segment of OVCF augmented by PKP; (3) patients voluntarily participated in the survey and understood the content of the study. The exclusion criteria were as follows: (1) presence of more than two recent vertebral fractures; (2) previous osteoporotic fractures; (3) neurological complications; (4) mental illness or a family history of mental illness; (5) spinal malignancy; (6) new osteoporotic fractures occurred during follow-up periods.

Patients in the CG were treated conventionally, and those in the IG were treated according to the ERAS protocol. The implementation of the ERAS protocol can be referenced by the expert consensus edited by our department in chief [16]. It included preoperative patient education with verbal and video information, preventive analgesia, an appropriate anesthetic plan, postoperative pain control, early mobilization, guidance on rehabilitation exercise, and more comprehensive care. Besides, we have enhanced patient engagement in ERAS by involving patients from the beginning and encouraging them to tell us about their ideas and needs so that patients could become knowledgeable partners in their recovery. Patients were administered a survey six times: at admission, postoperative follow-up at 1 week, and 1, 3, 6, and 12 months. The study protocol was approved by the Ethics Committee of The First Affiliated Hospital of Soochow University.

### Patient Health Questionnaire-9 (PHQ-9)

**Fig. 1:**
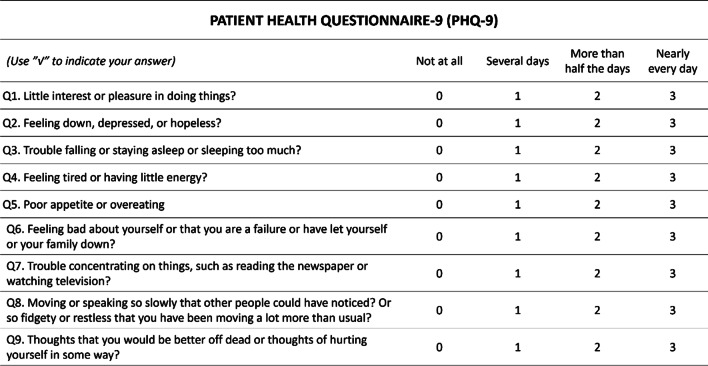
The rating scale of PHQ-9

The PHQ-9 is compiled based on the symptomatic criteria of depression in DSM-IV (Diagnostic and Statistical Manual of Mental Disorders, 4th Edition), which is a validated 9-item self-administered questionnaire that evaluates for depression [17] (Fig. 1). The scale includes 0–3 points to each entry (“not at all” = 0, “several days” = 1, “more than half the days” = 2, “nearly every day” = 3), and the total score is 27 points. A score of 0–4 indicated minimal depression, 5–9 mild, 10–14 moderate, 15–19 moderately severe, and 20–27 severe.

### Generalized anxiety disorder-7 (GAD-7)

**Fig. 2:**
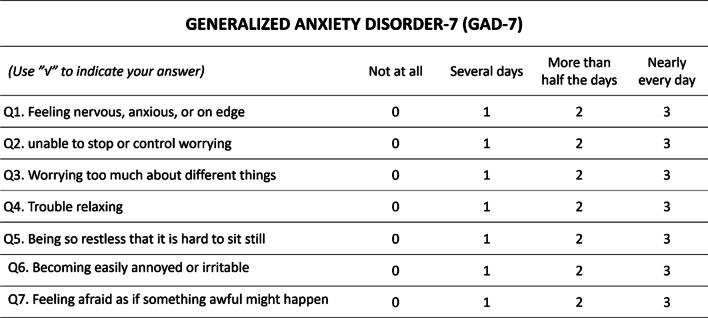
The rating scale of GAD-7

The GAD-7 is prepared based on the DSM-IV anxiety symptomatic Criteria, which is a validated 7-question scale designed to detect the presence of anxiety [18] (Fig. 2). The questionnaire includes 0–3 points for each item, with a total score of 21 points. The higher the score, the more functional impairment one has due to anxiety with minimal effect rated at a score of 0–4, mild 5–9, moderate 10–14, and severe 15–21.

### Statistical methods

The statistical analysis was performed with SPSS 23.0, the measurement data was expressed as mean ± standard deviation, and the count data was expressed as a percentage (%). To compare discrete variables, the chi-square test was used. The comparison of data from two groups was made by the independent sample t-test. One-factor repeated-measures analysis of variance was used to detect significant changes in the score of PHQ-9 and GAD-7 at adjacent follow-up time points in the two groups. Analysis of variance (ANOVA) was applied to compare the differences of each item of questionnaires at each period. *p* values < 0.05 were considered statistically significant.

## Results

Each number of cases excluded by the excluded criteria from one to six was 28, 40, 15, 10, 3, 43 and 15, 32, 19, 14, 7, 22 in the CG and in the IG respectively. A total of 323 patients in the CG and 306 patients in the IG were enrolled. Among them, 42 patients and 55 patients separately did not complete the survey for the follow-up periods. Eventually, 281 patients in the CG and 251 patients in the IG were included in this study. The background characteristics of the two groups were similar (Table 1).

**Table 1:** The background characteristics of the groups

Parameters	CG (n = 281)	IG (n = 251)	*p* value
Gender			
Male	229	211	0.434
Female	52	40	0.434
Age	66.30 ± 6.68	67.47 ± 7.66	0.062
VAS	7.15 ± 1.16	7.29 ± 0.96	0.126
Disease course	5.33 ± 5.07	5.82 ± 4.61	0.240
Marital status			
Married	213	196	0.532
Single, divorced or widowed	68	55	0.532

**Fig. 3:**
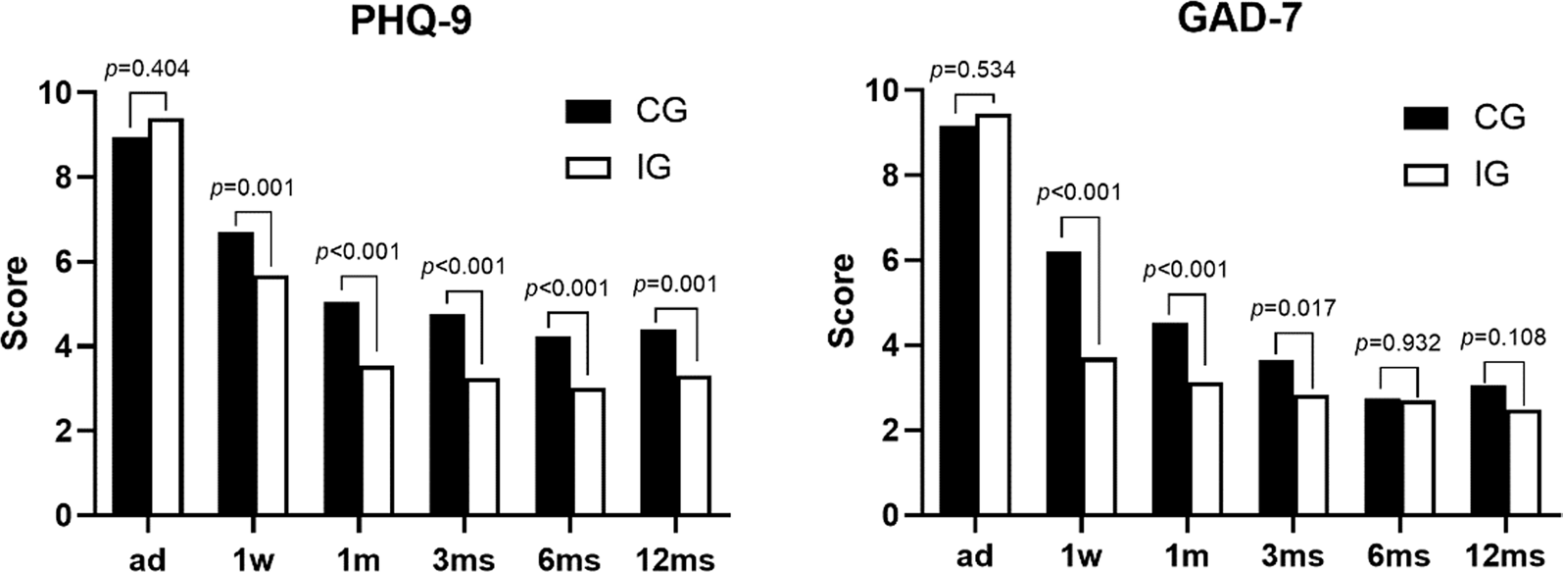
Mean scores of PHQ-9 and GAD-7 comparison between the control group (CG) and the intervention group (IG) at admission and follow-up periods (ad = admission; 1w = one week; 1 m = one month; 3 ms = three months; 6 ms = six months; 12 ms = twelve months)

When at admission, the PHQ-9 (CG: 8.96 ± 5.98, IG: 9.40 ± 6.10, *p* = 0.404) and GAD-7 (CG: 9.17 ± 4.87, IG: 9.45 ± 5.67, *p* = 0.534) scale score were no differences between the two groups. One week after surgery, the PHQ-9 scale score of the IG (5.67 ± 3.15) was statistically better than the CG (6.71 ± 3.63) (*p* = 0.001); the same results were observed in the subsequent follow-up periods, 1 month (CG:5.04 ± 3.84, IG:3.53 ± 2.46, *p* < 0.001), 3 months (CG: 4.76 ± 5.43, IG: 3.25 ± 2.33, *p* < 0.001), 6 months (CG: 4.24 ± 4.81, IG: 3.02 ± 2.21, *p* < 0.001) and 12 months (CG: 4.41 ± 4.70, IG: 3.31 ± 2.33, *p* < 0.001). The GAD-7 scale score showed no differences between the two groups at 6 months (CG: 2.74 ± 4.78, IG: 2.71 ± 3.61, *p* = 0.932) and 12 months (CG: 3.04 ± 4.26, IG: 2.49 ± 3.64, *p* = 0.108), while at 1 week (CG: 6.91 ± 3.85, IG: 3.71 ± 2.61, *p* < 0.001), 1 month (CG: 4.53 ± 4.31, IG: 3.13 ± 3.49, *p* < 0.001), 3 months (CG: 3.65 ± 4.11, IG: 2.82 ± 3.92, *p* = 0.017), the IG were significantly better (Fig. 3).

**Fig. 4:**
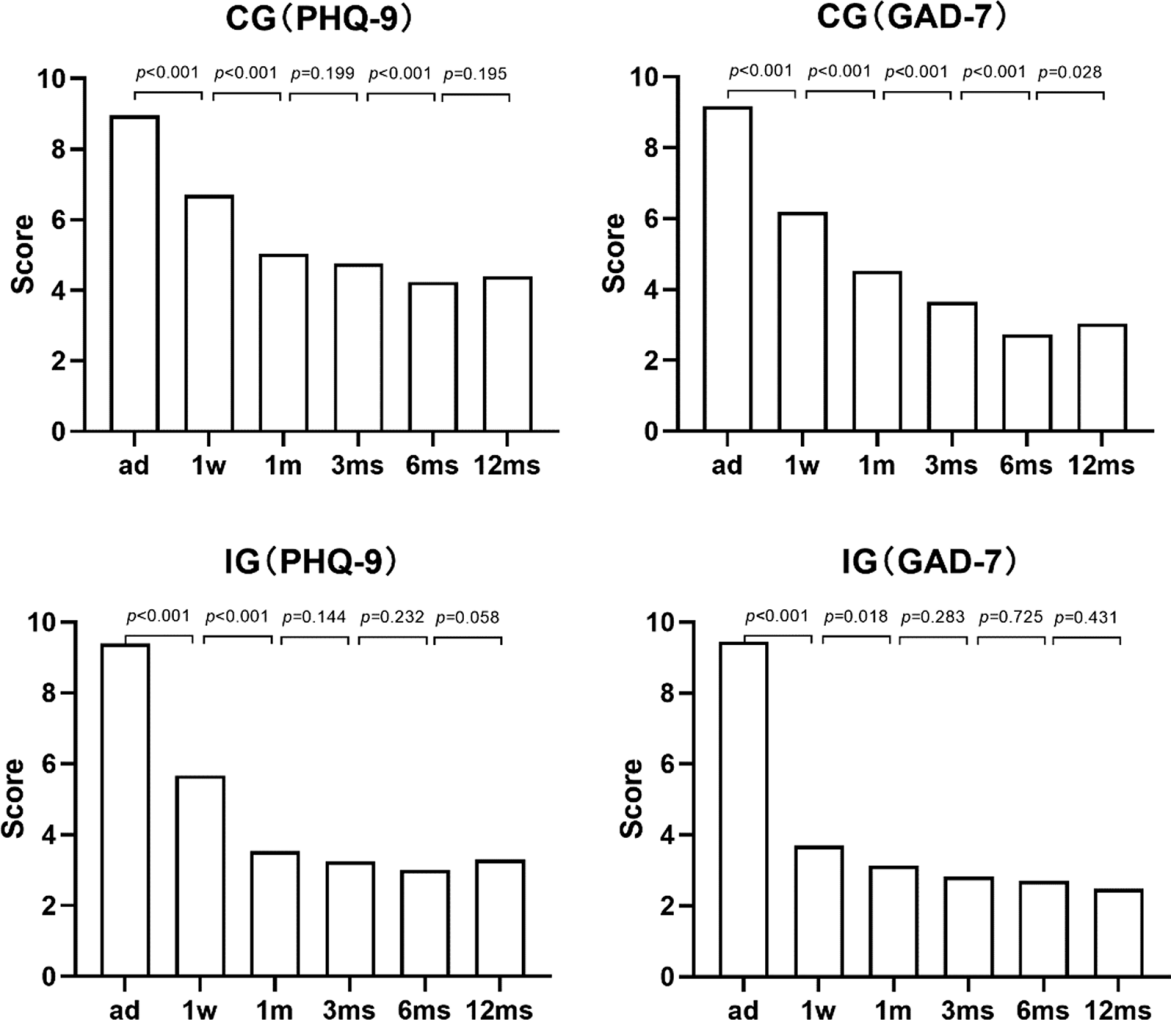
Mean scores of PHQ-9 and GAD-7 comparison between every period of the control group (CG) and the intervention group (IG)

When compared the scores of two adjacent follow-up time points, there were no differences between 1 and 3 months (*p* = 0.199), 6 months and 12 months (*p* = 0.195) of PHQ-9 in the CG, while at admission and 1 week, 1 week and 1 month, 3 months and 6 months of PHQ-9 and each one of GAD-7 was significantly different (*p* < 0.05). In the IG, the scores of PHQ-9 and GAD-7 were statistically better at 1 week than at admission (*p* < 0.05), 1 month than 1 week (*p* < 0.05), and showed no significant difference compared with it at next three months (Fig. 4).

**Fig. 5:**
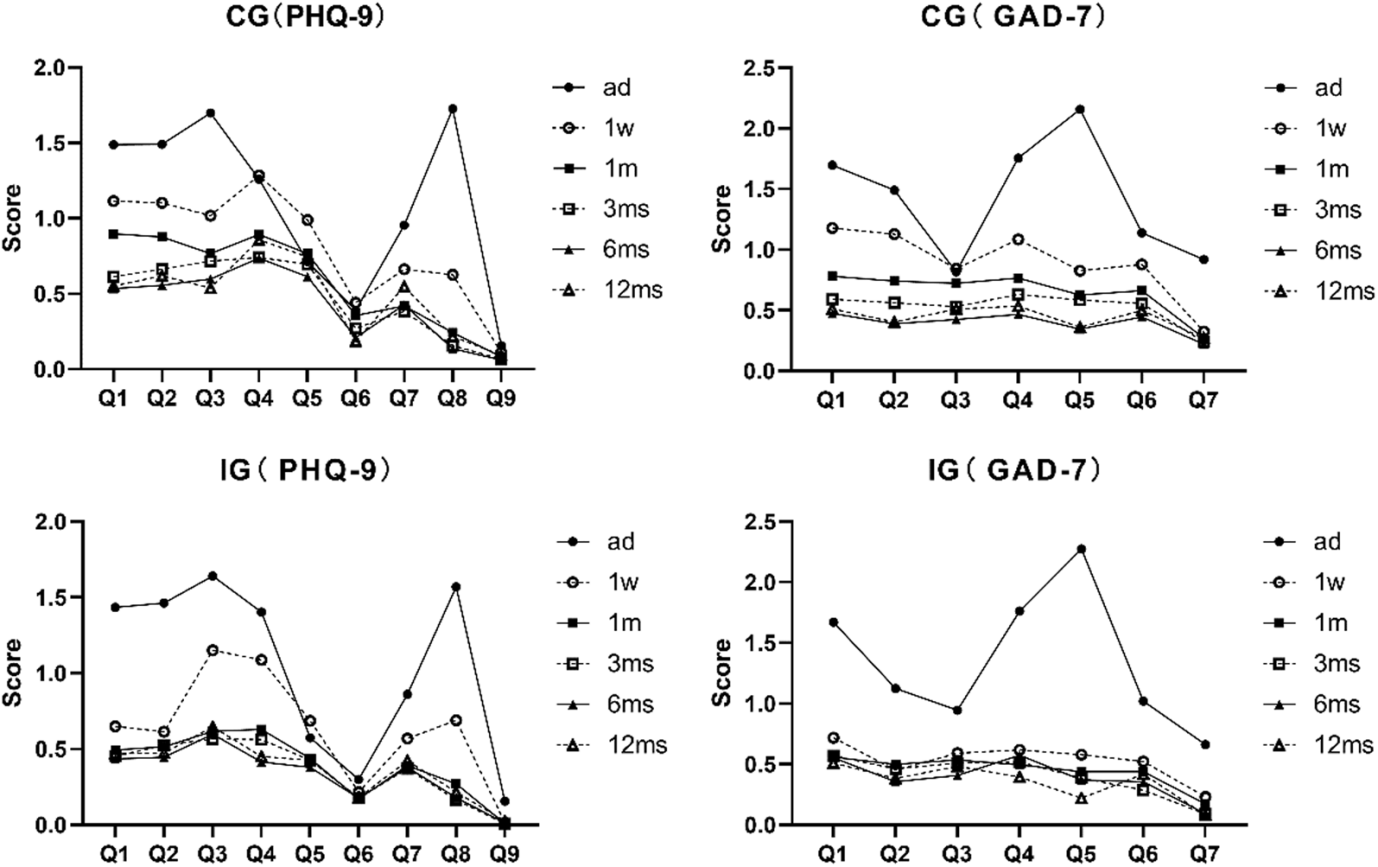
Mean scores of each item of PHQ-9 and GAD-7 at each period of the control group (CG) and the intervention group (IG)

The difference could be discovered when comparing the mean scores of each item of PHQ-9 and GAD-7 in each period of the CG and the IG, except for question 9 of PHQ-9 in the CG (*p* = 0.056), which referred to the act of committing suicide or harming oneself. Most of the items showed a trend of improvement, obviously question 1–4 and question 8 of PHQ-9, question 1–2, and question 4–5 of GAD-7 (Fig. 5).

**Fig. 6:**
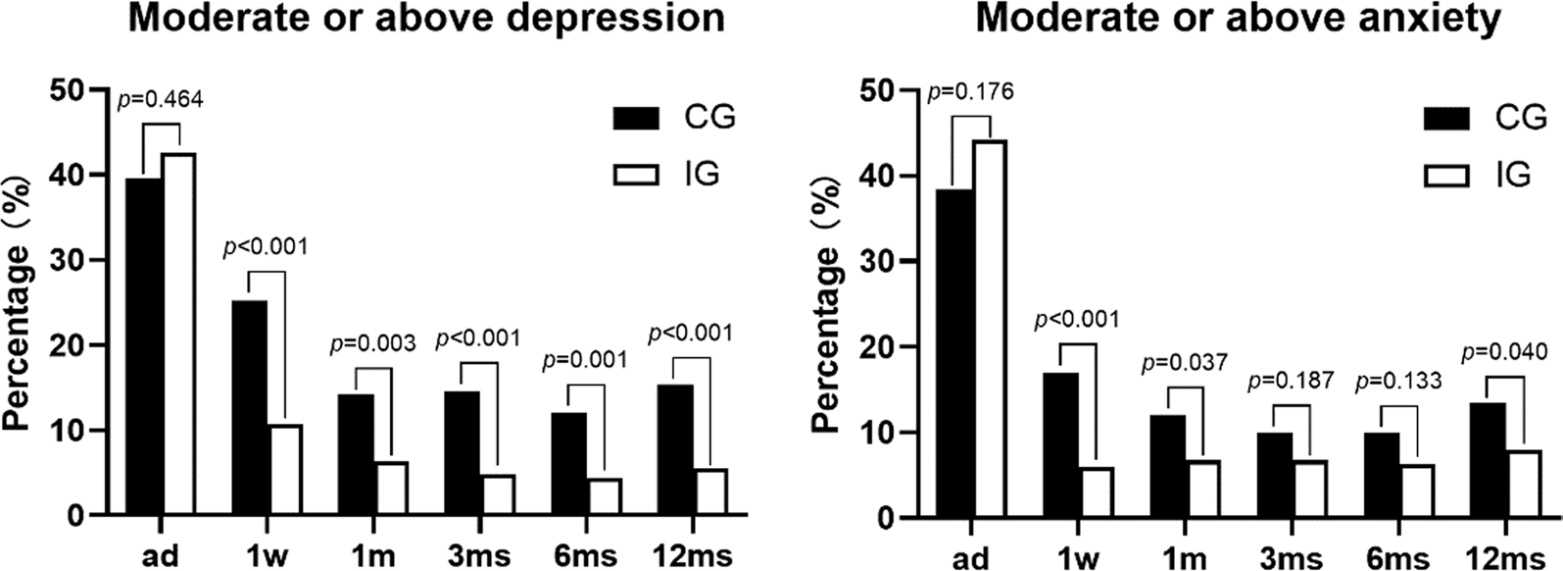
The percentage of patients with moderate or above depression or anxiety in the control group (CG) and the intervention group (IG)

The percentage of patients with moderate or above depression or anxiety did not differ at admission between the two groups. But the follow-up periods after surgery, fewer people had moderate or above depression in the IG (*p* < 0.01). The odds ratio (OR) was respectively 0.410 (0.224, 0.753), 0.357 (0.220, 0.577), 0.294 (0.151, 0.573), 0.333 (0.165, 0.672), 0.327 (0.174, 0.613) from 1 week to 12 months. When at 1 week (*p* < 0.001), OR = 0.528 (0.287, 0.970), 1 month (*p* = 0.037), OR = 0.309 (0.168, 0.566), and 12 months (*p* = 0.040), OR = 0.554 (0.313, 0.980), the percentage of patients with moderate or above anxiety significantly decreased in the IG, while there were no differences between the other two months (Fig. 6).

## Discussion

ERAS protocols represent a feature of contemporary perioperative medicine and have been adopted by various surgical department [13]. The implementation of ERAS rested on a multidisciplinary team working together and a multimodal approach to optimize the perioperative measures to minimize stress, accelerate recovery and reduce complications [13, 19]. To date, the outcomes of ERAS have been assessed by traditional clinician-based assessments of functional improvement after surgery, such as length of hospital stay, hospitalization expense, and complication rates [20–22]. However, few studies have been implicated in psychological intervention. As mentioned above, psychological factors play an essential role in the prognosis of surgery; hence we studied depression and anxiety by comparing ERAS and conventional protocols in patients undergoing PKP surgery.

Our study showed that the ERAS protocol reduced the incidence of moderate or above depression and anxiety and the degree of depression and anxiety more effectively. Moreover, patients could soothe anxiety disorders and depression disorders faster and maintain psychological stability during the follow-up periods in the ERAS group. The reason may be that we carried out preoperative patient education and gave postoperative rehabilitation guidance, so patients could coexist with the disease easier, which verified the view of Gillis et al. [14]. While in the conventional treatment group, patients suffered postoperative psychological fluctuations due to a lack of awareness of the disease, especially about restoring physical function and possibly future fractures. On the other hand, due to the fracture-related dysfunction and pain, the scores of individual items were significantly high, particularly the questions involving the quality of sleeping and the ability to walk and sit. Those were improved considerably in both groups after surgery.

In our study center, the ERAS program is summarized as “start with evaluation and end with evaluation”, which means each patient should be evaluated individually and comprehensively, including cardiorespiratory fitness, nutritional status, pain severity level, and risk of deep vein thrombosis. As to psychological intervention, perioperative education is an essential part. The medical staff and the patients will discuss the disease cause, the treatment plan, how long they can expect to be in the hospital, discharge criteria, rehabilitation program, and subsequent visit time. Besides, preventive analgesia is promoted because patients with intense pain are at greater risk of anxiety and depression [15]. The principle of analgesia is multimodal analgesia and priority to non-steroidal anti-inflammatory drugs (NSAIDs). Insomnia is impacted by pre-existing pain, anxiety, and depression, also the subsequent onset of them [23]. So we increasingly pay attention to improving patients’ quality of sleep. Improving sleep surroundings and providing guidance on sleep hygiene have been given, and hypnotics would be applied appropriately when needed.

As to the assessment of depression and anxiety, we chose the scale of PHQ-9 and GAD-7 due to their simplicity, universality, and reliability [24, 25]. When patients were admitted to the hospital, the medics took an evaluation about depression and anxiety state. If a patient suffered from moderate to severe depression or anxiety, we would ask psychologists for further assessment and intervention except for perioperative education. In this way, patients who presented significant symptoms could receive timely psychological intervention and achieve the best surgical outcomes.

Depression and anxiety can be caused by various factors, such as family income, education background, medical insurance, living condition, family support, and so on. A limitation of this study was that we did not take all these factors into account and analyze the correlations. In addition, our study was not a randomized control, leading to a loss of credibility. Besides, the extrapolation of our findings is limited because our data were collected from a single center, and we hope that our subsequent trials will go further.

## Conclusions

In summary, this cohort study revealed that the PKP procedure with ERAS protocol to treat patients with OVCF had a better effect on relieving the degree of postoperative anxiety and depression. So we support the use of ERAS in this surgery.

## Data Availability

All the data are available if qualified authors apply for them.

## Abbreviations

PKP: Percutaneous kyphoplasty
ERAS: Enhanced recovery after surgery
OVCF: Osteoporotic vertebral compression fracture
